# Center for stroke disparities solutions community- based care transition interventions: study protocol of a randomized controlled trial

**DOI:** 10.1186/s13063-015-0550-3

**Published:** 2015-01-27

**Authors:** Penny H Feldman, Margaret V McDonald, Melissa A Trachtenberg, Antoinette Schoenthaler, Noreen Coyne, Jeanne Teresi

**Affiliations:** Center for Home Care Policy and Research, Visiting Nurse Service of New York, 107 East 70th Street, 10021 New York, NY USA; Center for Home Care Policy and Research, Visiting Nurse Service of New York, 5 Penn Plaza, 12th floor, 10001 New York, NY USA; Center for Healthful Behavior Change, Department of Population Health, NYU School of Medicine, 227 East 30th Street, 634, 10016 New York, NY USA; Columbia University Stroud Center and New York State Psychiatric Institute, Research Division, Hebrew Home at Riverdale, 5901 Palisade Avenue, 10471 Bronx, NY USA

**Keywords:** Care transitions, Home health, Stroke, Hypertension, Blood pressure, Health disparities, Trial design

## Abstract

**Background:**

Racial and ethnic disparities persist in stroke occurrence, recurrence, morbidity and mortality. Uncontrolled hypertension (HTN) is the most important modifiable risk factor for stroke risk. Home health care organizations care for many patients with uncontrolled HTN and history of stroke; however, recurrent stroke prevention has not been a home care priority. We are conducting a randomized controlled trial (RCT) to compare the effectiveness, relative to usual home care (UHC), of two Community Transitions Interventions (CTIs). The CTIs aim to reduce recurrent stroke risk among post-stroke patients via home-based transitional care focused on better HTN management.

**Methods/Design:**

This 3-arm trial will randomly assign 495 black and Hispanic post-stroke home care patients with uncontrolled systolic blood pressure (SBP) to one of three arms: UHC, UHC complemented by nurse practitioner-delivered transitional care (UHC + NP) or UHC complemented by an NP plus health coach (UHC + NP + HC). Both intervention arms emphasize: 1) linking patients to continuous, responsive preventive and primary care, 2) increasing patients’/caregivers’ ability to manage a culturally and individually tailored BP reduction plan, and 3) facilitating the patient’s reintegration into the community after home health care discharge. The primary hypothesis is that both NP-only and NP + HC transitional care will be more effective than UHC alone in achieving a SBP reduction. The primary outcome is change in SPB at 3 and 12 months. The study also will examine cost-effectiveness, quality of life and moderators (for example, race/ethnicity) and mediators (for example, changes in health behaviors) that may affect treatment outcomes. All outcome data are collected by staff blinded to group assignment.

**Discussion:**

This study targets care gaps affecting a particularly vulnerable black/Hispanic population characterized by persistent stroke disparities. It focuses on care transitions, a juncture when patients are particularly susceptible to adverse events. The CTI is innovative in adapting for stroke patients an established transitional care model shown to be effective for HF patients, pairing the professional NP with a HC, implementing a culturally tailored intervention, and placing primary emphasis on longer-term risk factor reduction and community reintegration rather than shorter-term transitional care outcomes.

**Trial registration:**

ClinicalTrials.gov NCT01918891; Registered 5 August 2013.

## Background

Despite improvements in reducing stroke first-ever occurrence, mortality and recurrence over the past few decades [1-3], racial and ethnic disparities persist. Risk of having a first stroke for blacks is nearly twice as high as for whites, and blacks are more likely to die following a stroke than are whites [4]. The risk of stroke for Hispanics falls between that of whites and blacks [4]. A similar pattern is seen in recurrent stroke, with blacks and Hispanics having higher recurrence rates than whites [5-8]. Approximately, one quarter of the strokes that occur each year are in people who had a previous stroke [4]. Compared to first strokes, recurrent strokes are associated with higher mortality, greater disability, and greater health care costs [9,10].

While reasons for racial and ethnic disparities in stroke and stroke recurrence are complex, numerous studies have demonstrated that blacks and Hispanics have disproportionately higher rates of stroke risk factors than whites, including uncontrolled hypertension (HTN), poorer diabetes control, and higher rates of hyperlipidemia [11,12]. HTN is a particularly important factor as it has been found repeatedly to be a predictor of stroke and stroke recurrence in the black and Hispanic populations [11-15] and has been identified as the single most important modifiable stroke risk factor [14,16,17]. Multiple studies and clinical guidelines have been published indicating the benefits of HTN control on reducing stroke risk [18-20]. Authors of a review of prospective cohort studies and a meta-analysis of over 40 randomized controlled trials found that in both sets of studies each 10 mmHg lower systolic blood pressure (SBP) was associated with a one third reduction in stroke risk [19].

Despite the benefit of HTN control, a large treatment gap exists between the medical and behavioral regimens recommended for recurrent stroke prevention and actual post-stroke care received by patients [21]. The American Heart Association and American Stroke Association (AHA/ASA) jointly issued a statement in 2011, acknowledging racial and ethnic disparities in different patients’ stroke experiences, including access to and quality of care [22]. Our study seeks to address disparities and service gaps by identifying and intervening with a particularly vulnerable population - black and Hispanic patients who have a history of stroke, currently have uncontrolled HTN and are patients transitioning in and out of post-acute home health care services. Data from the National Home and Hospice Survey, which is conducted by the Centers for Disease Control, indicate that 7% of home care patients have a history of cardiovascular disease and 41% have HTN [23]. Each day there are approximately 1.5 million patients enrolled in home health care [23].

Home care not only constitutes an unexplored environment to intervene to prevent stroke recurrence, it also presents an opportunity to do so in the context of care transitions, a time when patients are particularly susceptible to adverse events [24,25]. This Community Transitions Intervention (CTI) study aims to assess the comparative effectiveness, relative to usual home care (UHC), of adding a nurse practitioner (NP)-delivered transitional care intervention or a transitional care intervention delivered by an NP + health coach (HC). Clinical trials of NP-delivered transitional care interventions have shown them to be effective in improving outcomes of heart failure (HF) patients [24]. Trials of community health worker and coaching interventions have shown significant potential to improve blood pressure (BP) control [26-29]. However, NP transitional care alone or combined with HC support has never been tested as a vehicle for risk reduction among post-stroke patients.

Both intervention arms have a three-fold focus: 1) linking patients to continuous, rapidly responsive preventive and primary care, 2) increasing patients’/caregivers’ ability to manage a culturally and individually tailored BP reduction plan, and 3) facilitating the patient’s reintegration into the community after home health care discharge. The CTI is significant in that it targets care gaps affecting a particularly vulnerable population characterized by persistent stroke disparities (blacks/Hispanics). It is innovative in that it adapts for stroke patients established transitional care models shown to be effective for HF patients [26,30], pairs the professional NP with a community HC and gives emphasis to longer-term risk factor reduction and community reintegration rather than short-term transitional care outcomes.

## Methods/Design

The study is designed as a three-arm RCT so that we can test individually the comparative effectiveness of adding: a) just an NP CTI, or b) an NP + HC CTI to routine care -–that is UHC. The primary hypothesis is that either intervention will be more effective in reducing blood pressure than the UHC condition. There is no hypothesis regarding the relative merits of one of the intervention arms over the other. Thus, the study is powered to compare the effects of each intervention (NP and NP + HC) to UHC. However, in sensitivity power calculations for non-linear change models, scenarios with different effect sizes associated with each intervention will be examined.

### Setting: study site

**Figure 1:**
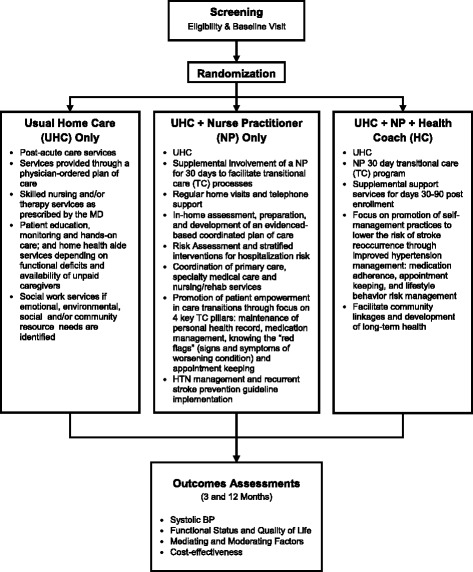
**Community Transitions Intervention study design.**

The study is being conducted in the post-acute care division of a large, urban, nonprofit, Medicare-certified home health organization. The majority of post-acute care stroke patients are admitted to home care immediately after a hospital discharge; others come through community referrals (primarily physicians). All patients in the study (regardless of study arm) will receive UHC (see Figure 1). The study was approved by the Visiting Nurse Service of New York’s (VNSNY’s) institutional review board (reference #I12-004).

### Control condition: usual home care

UHC consists of: a physician-ordered plan of care; skilled nursing and/or therapy services as prescribed by the medical doctor; patient education, monitoring and hands-on care; home health aide services depending on functional deficits and availability of unpaid caregivers; and social work services if emotional, environmental, social and/or community resource needs are identified. All nurses and therapists use tablet computers, electronic messaging and an electronic health record (EHR); and have access to a decision support system. The decision support system includes a discretionary module on care of stroke patients that outlines information to teach the patient/caregiver (for example, manifestations/causes/contributing factors to stroke, behaviors to manage a stroke, and symptoms/complications to seek emergency care). The module also gives nurses information on helping patients manage motor/sensory/cognitive deficits. The module emphasizes stroke recovery/rehabilitation rather than secondary prevention.

### Interventions

#### Nurse Practitioner (NP) Arm

The study’s NPs are employed by a professional practice group affiliated with the parent home health organization. The practice group provides primary and specialty medical services and care coordination and can bill third parties. The intervention, which complements UHC, builds on an existing 30-day transitional care program established in the practice group. Program components are modeled after two evidence-based models: 1) the Transitional Care Model (TCM) developed and validated for HF patients [26], which uses an advanced practice nurse for intervention activities, and 2) the Four Pillars of the 30-day Care Transitions Intervention® (CTI) [30], which emphasizes patient self-management. Both models have demonstrated effectiveness in reducing hospital readmissions [25,31]. Because the intervention commences only after a patient is admitted to home care, we have omitted the element that calls for visiting the patient in the hospital before discharge. This element has been omitted in other tests of the TCM [32]. We also have modified the traditional TCM in at least two other ways: 1) to allow for the inclusion of at-risk eligible patients referred to home care from community physicians, and 2) to increase the emphasis on stroke recurrence prevention - specifically on reducing SBP. We incorporated and combined the goal of improved transitional care with the goal of effective chronic disease self-management. The program length of 30 days was chosen because it is the allowable Medicare reimbursable timeframe for transitional care services and thus should contribute to the sustainability of the model after the grant period ends.

With intervention patients the NP is responsible for: 1) conducting a comprehensive health assessment; 2) establishing linkages with the patient’s physicians (specialists and primary care) and UHC nurse; 3) working with the physicians to specify recommended medication and behavioral regimens; 4) coordinating with the patient and the patient’s caregivers to formulate patient goals; 5) tailoring the treatment plan to the patient’s preferences and circumstances; and 6) providing collaborative problem solving and self-management support to the patient and caregivers. The NP patient-contact protocol includes: 3 in-home visits, 3 patient-caregiver telephone calls, and a varied amount of inter-professional collaboration calls over the 30-day intervention period. Between the in-home visits, the patient-caregiver calls provide the opportunity for the NP to review with patients/caregivers their medical appointments, test results and medication issues and to reinforce progress with lifestyle management. The NP also may attend a joint visit to the patient’s primary care physician for patients who are having difficulty with their treatment plan. Additional resources that the NP provides and discusses with the patient/caregiver are a transitional care booklet that includes worksheets to be used to develop a personal health record and an (AHA/ASA) packet ‘*Understanding and Controlling your High Blood Pressure*’ educational guide. These guides are provided in English or Spanish as preferred by the patient.

#### NP + health coach (HC) Arm

This arm, like the NP-only arm, complements UHC. It is designed so that all patients receive the intensive 30-day NP intervention as described above plus an additional 60 days of collaboration and self-management support from a specially trained HC. The HC joins the NP at the third patient intervention visit and subsequently provides 3 in-home visits and 3 telephone calls for HC/patient-caregiver collaboration. The planned schedule of NP/HC contacts is outlined in Table 1.

**Table 1:** **Intervention staff-patient encounter schedule**

**Week**	**NP**	**HC**
1	Visit	
	Call	
2	Visit	
3	Call	
4	Call	Visit
	Visit	
5		Call
6		
7		Visit
8		Call
9		
10		Call
11		
12		Visit

Notes: HC = health coach; NP = nurse practitioner.

Home health aides and other community members who have similar racial and ethnic backgrounds as the target patient population have been engaged and trained as community health coaches for this study. The training curriculum was based on the principles of adult learning theory, the core competencies identified by the National Community Health Advisor study [33], and the Training Curriculum for Health Coaches developed by Bodenheimer at UCSF Center for Excellence in Primary Care [34]. The coaches were trained to use motivational interviewing strategies (for example, setting the agenda, reflective listening, building motivation for change, goal setting) [35] to facilitate relationship building, assist with problem-solving, and to promote behavior change. Training was led by one of the co-investigators (AS), who is a member of the Motivational Interviewing Network of Trainers (MINT).

The key elements of the HC role are: 1) communication with patients and their informal caregivers to promote recurrent stroke prevention awareness, education, and understanding of risk factors and recommended medical and lifestyle regimens; 2) collaborative interactions to improve self-management; and 3) navigation and networking to facilitate patients’ social and physical integration into the community for lasting change. The HC uses the AHA/ASA educational guide distributed by the NP, as noted above, to support his or her efforts in working with the patient on setting behavior change goals.

### Intervention fidelity monitoring protocol

Monitoring activities are outlined below:

1. Review NP adherence to intervention protocol
  Primary monitoring
  - #/% of 6 encounters planned; track #/% of 3 in-home visits and #/% of 3 telephone calls
  - # of encounters above the 6 planned
  - #/% of cases in which HTN management was addressed
  - #/% of cases joint NP-HC visit was completed, when appropriate
    Supplemental monitoring
  - #/% of patients who have a joint NP-MD visit
  - # of collaborative-coordination calls per patient
2. Review health coach adherence to intervention protocol
  Primary monitoring
  - #/% of 6 encounters planned; track #/% of 3 in-home visits and #/% of 3 telephone calls
  - # of encounters above the 6 planned
  - #/% of cases in which at least 1 HTN self-management goal was established
  - Completion rate of requested audio-recordings
  - #/% of audio-recordings in which motivational interviewing principles were demonstrated
    Supplemental monitoring
  - #/% of patients who receive a community resource referral

Abbreviations; HC = health coach; HTN = hypertension; MD = doctor of medicine, NP = nurse practitioner.

NP and HC intervention staff enter information on each of their patient encounters in the EHR of the transitional care program. Patient-level and aggregate reports, generated for regular review by the principal investigator, the project manager and the field coordinator, include data on the number and types of encounters by each discipline, time of each encounter, and number of collaborative-coordination calls. Data about HTN management interventions and goal setting/achievement is also being extracted, tracked, and monitored. NPs are involved in regular case reviews. The health coaches are audio-recording encounters with 20% of their patients. These sessions are being evaluated using the Behavior Change Counseling Index (BECCI) [36]. The BECCI is an instrument designed for trainers to score practitioners’ use of behavior change counseling strategies in their patient encounters. Coaches receive regular feedback on their intervention activities by the project manager and two MINT trainers.

### Eligibility and inclusion/exclusion criteria

A total of 495 black and Hispanic patients (≥21 years of age) who have had a first time or recurrent stroke (ischemic or hemorrhagic) or transient ischemic attack (TIA) and have uncontrolled SBP will be recruited: 165 randomized to each group. To be eligible a patient also needs to be English or Spanish speaking and have a telephone to use for intervention encounters. Patients are excluded if they have a clinical condition that may require specialized HTN management (for example, end stage renal disease, severe HF). Additional inclusion/exclusion criteria are listed in Table 2.

**Table 2:** **Study participant eligibility criteria**

**Inclusion criteria**	**Exclusion criteria**
▪ Average screening systolic BP ≥ 140 mmHg on 2 screening visits within 7 days of each other	▪ Average screening systolic BP ≥ 200 mmHg or average diastolic BP ≥ 120 mmHg (on 3 consecutive visits)
▪ Recently admitted to the home care post-acute care program	▪ Dialysis^a^
	▪ End stage renal disease^a^
▪ 21 years of age or older	▪ Kidney transplant^a^
▪ Black and/or Hispanic	▪ Severe heart failure^a^
▪ Speaks English or Spanish	▪ Significant cognitive impairment. Unable to provide informed consent, accurate self-report, and/or unable to participate effectively in intervention
▪ History of stroke (ischemic or hemorrhagic) or transient ischemic attack	
▪ HTN diagnosis	▪ Patients with upper arm circumference ≥ 38 cm. At this dimension our blood pressure cuffs become inaccurate
▪ Resides in the study catchment area	
▪ Has a telephone (in order to participate in intervention phone sessions)	

Notes: BP = blood pressure; HTN = hypertension.

^a^Conditions require very specialized care of HTN, including different medications and dietary/physical activity recommendations.

### Procedures

#### Screening

Initial identification is through a review of EHR data for new admissions into the organization’s post-acute care program. Electronic records were used to initially identify potential patients using a variety of *International Classification of Diseases, version 9* (ICD-9) codes for post-stroke care (with the vast majority of patients coming in with a code of 438.xx - late effects of cerebrovascular disease). Confirmation of the history of stroke or TIA was completed through patient self-report. After patients are enrolled, they are asked to sign a release for their hospital discharge summaries so the investigator group can retrieve additional information on their most recent stroke. Confirmation of additional eligibility criteria requires that the patient pass: 1) a telephone eligibility screen, 2) an initial in-home BP check, and 3) a confirmatory BP check within 7 days of the initial check. These three steps are conducted by specially trained study interviewers who receive extensive didactic, role play, and field training prior to independent deployment.

On the first BP screening visit, three readings are taken simultaneously on both arms using a validated, automated oscillometric BP device (Microlife Watch BP, Golden, CO, USA) and an average is provided. If a patient’s SBP is 140 mmHg or greater on either arm, the patient is scheduled for a follow up in-home BP screening within 7 days. On the second visit, BP is re-measured three times on the arm that had the higher SBP reading (the ‘dominant arm’). If the average SBP is again ≥ 140 mmHg, the level that meets our study criteria, the interviewer proceeds with recruitment, the informed consent process. Patients with a systolic BP ≥ 200 mmHg or diastolic BP of ≥ 120 mmHg at the screening visits are directed to receive immediate medical attention. If the patient is willing, an additional BP screening visit will be scheduled for at least 48 hours after the critical BP screen visit to determine potential eligibility. If the patient’s BP continues to be critical, they will be advised to seek immediate medical attention and will not be enrolled in the study.

#### Informed consent

If the patient has a SBP of 140 mmHg or greater at both screenings then the interviewer proceeds with the informed consent process. The interviewer provides the patient with the hardcopy consent and among other things describes the three potential study groups, the content of the interventions, the baseline and follow up interview schedule, the sharing of information with others on the study team and that involvement is voluntary. The interviewer confirms that the patient understands what they are consenting to, and if the patient agrees two consents are signed: one for study files and one for the patient. Informed consent is obtained from each participant.

#### Baseline assessment

Once a consent form is signed, the interviewer proceeds directly with the baseline assessment. The assessment is a structured interview, largely made of up self-report measures. (See Measurements section below).

#### Randomization and blinding

Following the baseline interview, subjects are randomized to one of three arms. Upon randomization to one of the intervention arms, the UHC nurse who is designated as the main nurse organizing and providing routine home health visits to the patient is sent a secure Email message indicating the patient’s involvement in the CTI. The Email outlines the objectives of the program and provides information on how to contact the transitional care NP involved in the case. As necessary/appropriate, the NP contacts the UHC nurse, just as the NP contacts the physician or any other health care provider of the patient as medically necessary. Throughout the course of the study, field interviewers collecting baseline and outcome data are blinded to the patient assignment group.

#### Anticipated study characteristics

The gender distribution is expected to reflect the home care population with the targeted clinical conditions. Because the goal of study is to determine whether the interventions will improve blood pressure control in racial minorities after stroke, the participants in this study will be self-identified as black or Hispanic. Based on available organization data and prior studies, the enrolled sample is expected to be 65% black/35% Hispanic; 40% male/60% female; with a mean age of 67 years.

### Measurements

#### Data sources

To achieve our specific aims, we will make use of data from: 1) patient assessments and interviews conducted during the initial phone screen, at baseline (enrollment in the study), and 3 and 12 months post-study enrollment; and 2) intervention cost data collected especially for this study. Information to be obtained is summarized in Table 3. Additionally, patient-level clinical and functional assessment data derived from OASIS, the nationally mandated home care Outcomes Assessment and Information Set will be used to assess potential differences in those participating in the study and those who declined. This will be used for discussion of the generalizability of our findings.

**Table 3:** **Information to be collected and used in analysis**

	**Baseline**	**3 Months**	**12 Months**
**Patient Characteristics**			
Basic sociodemographics: age, sex, race, ethnicity, preferred language to use, living situation, education, health literacy	X		
Marital status, employment status, income	X	X	X
Acculturation: place of birth, parent and grandparent place of birth, length of time in US	X		
Executive Cognitive Function (FAB)	X		
Stroke, HTN and hospitalization history; co-morbidities (modified Charlson)	X	X	X
**Clinical and functional outcomes**			
**Primary outcome**			
Blood pressure measurement	X	X	X
**Secondary outcomes**			
Cost-effectiveness	X	X	X
Health-related quality of life (EuroQol)	X	X	X
ADL/Functional Status (modified Barthel/Rankin for patient self-report)	X	X	X
Physical function (PROMIS®)	X	X	X
New cardiovascular events		X	X
**Exploratory outcome mediators**			
Knowledge of HTN management	X	X	X
Stroke literacy questions (Willey)	X	X	X
Adherence to regimen (Morisky)	X	X	X
Beliefs about medications (BMQ)	X	X	X
Linkage to PCP	X	X	X
Chronic condition management (PACIC)	X	X	X
General self-efficacy scale (Lorig)	X	X	X
Lifestyle management: diet/sodium intake (modified CATCH), physical activity (IPAQ), tobacco, alcohol and drug use, body mass index	X	X	X
Depression (PROMIS®)	X	X	X
Medication profile	X	X	X
**Organizational and system costs**			
Intervention costs: nurse practitioner and health coach time, patient educational material		X	X
Home care-related costs: nurse, therapy, social work, and aide home care visits		X	X
Overall medical costs: nights in hospital, ED visits, physician visits, medications		X	X

Notes: Barthel/Rankin [37,53]; FAB = Frontal Assessment Battery [54]; Charlson, modified self-report [55,56]; EuroQol [38]; PROMIS® [57]; Willey [58]; Morisky, 8-item version [59]; BMQ [60], PACIC = Patient Assessment of Chronic Illness Care, modified subscales [61]; Lorig [62]; CATCH [63]; IPAQ = International Physical Activity Questionnaire [64,65]; PROMIS® depression [66]; ED = emergency department; ADL = activities of daily living; PCP = primary care provider; HTN = hypertension.

### Primary outcome: change in systolic blood pressure

The primary outcome is change in SBP from baseline to 3 and 12 months. As with the baseline interview, patients are assessed with a validated, automated oscillometric BP device (Microlife Watch BP, Golden, CO, USA). The average of the BP measurements from the two enrollment screening visits will be used for comparative analysis to the follow up measurements. At the 3- and 12-month follow up evaluations, the BP will be measured by interviewers, blinded to group assignment, 3 times on the dominant arm identified at baseline and the average will be used for analysis.

### Secondary outcomes: cost-effectiveness, patient function, and quality of life

Costs will focus on direct costs including costs of the interventions, home care utilization, hospital and emergency department use, outpatient visits and medication regimens. Patient function and health-related quality of life will be assessed with a modified self-report Barthel Index [37] and the EuroQol [38]. See Table 3.

### Exploratory outcomes: moderators and mediators

Data are being collected so that moderators and mediators that may affect treatment outcomes can be explored. Moderators include race and ethnicity (that is black/Hispanic) differences and baseline HTN severity (for example, Stage 1 (SBP = 140 to 159 mmHg) versus Stage 2 (SBP ≥ 160 mmHg)). Mediators include changes in health behaviors (for example, diet, physical activity, weight loss, medication adherence) and antihypertensive medication intensification.

### Statistical analysis plan

#### Data analyses for primary outcome: SBP

The main hypothesis is that those assigned to the intervention groups will, on average, exhibit greater 3-month and 12-month decreases in SBP than those assigned to the usual care condition. The primary proposed analyses will use mixed random effects models, and a full information maximum likelihood (FIML) approach, with sensitivity analyses using generalized estimating equation (GEE) regression models. The change from pre- to post-treatment values of SBP will be modeled as functions of time, treatment and the interaction of time and treatment. The general longitudinal mixed effects model, using SAS PROC MIXED (SAS, Cary, NC, USA), will be used to model serial correlations and group heterogeneity in residual variances if needed. The intent-to-treat (ITT) analyses will permit all individuals with at least one observation to be included. If a non-linear pattern of change is observed, non-linear models will be used in sensitivity analyses.

Prior to analyses, baseline values of all variables will be examined in order to determine if any covariates require modeling due to imbalance among groups. Examination of baseline differences on key variables between completers and those lost to follow up will be conducted to inform about the nature of the missing data.

#### Power for SBP rate of change

Power was calculated, examining the rate of change, including all three waves in the analyses. The following assumptions were used: R = 0.90 (reliability); pooled σ = 20.75; δ = 5, 6, 7 (SBP point reduction per year in the intervention groups relative to the UHC group reduction); d = δ/σ (where δ is point reduction per year and d is Cohen’s d for the rate of change); ρ = 0.6 is the average correlation between baseline and follow up assessments; Tn = 3 time points (baseline, 3 month and 12 month). The formulas from Diggle, Liang and Zeger (1994) were used [39]: the power calculation demonstrates that under ITT, we can detect a rate of reduction in SBP equivalent to 5.40 mmHg which translates to relatively small effect sizes using Cohen’s d. It is also noted that the assumed standard deviation was conservatively posited to be quite large (pooled estimate of around 21). If a smaller standard deviation is observed, power will be greater. Thus, a sample size of 165 per group was proposed.

For the non-linear, longitudinal change sensitivity analyses [40], the assumptions were the same as above, and an endpoint reduction in SBP of 6 points was posited. Assuming a non-linear decline function and differential treatment effects, power was greater than 0.80 to detect small effects (for example, a 0.5 point reduction) in one intervention group at 3 months and medium to large effects (2.5 to 5 point reduction in SBP) in the second group at 3 months, with a net reduction of 6 points by study end in both groups.

## Discussion

The cumulative 5-year risk of having a recurrent stroke after a person’s first-ever stroke is over 30%, and the 30-day fatality risk associated with secondary strokes has been reported to be 40 to 50%, which is significantly higher than first stroke fatality rates [41,42]. Blacks and Hispanics have higher risk of recurrence than whites [5,7,8]. A contributor to this ongoing risk of stroke survivors is ineffective recurrent stroke preventive interventions. Our interventions are designed to specifically address HTN, a major risk factor for strokes and recurrent strokes, and are built on NP and HC models found to be successful in addressing other clinical conditions and established in other care settings. The NPs are positioned to complement UHC services by assessing and addressing hospitalization risk factors that are common during transitional care periods, while also providing a specific HTN management intervention geared toward preventing stroke recurrence. Both the NP and NP + HC interventions expand on the usual transitional care focus, which is on hospital discharges and preventing rehospitalization, by focusing on longer-term stroke risk prevention, community provider connections to facilitate long-term chronic care management, and reintegration into the community.

Our interventions were informed by models that showed promise in other settings. Focus groups and individual interviews were also conducted with black and Hispanic post-stroke patients who had received home care and their caregivers to further inform our intervention approach along with our enrollment efforts. During these focus groups, post-stroke patients expressed fear of research and lack of trust in researchers. They suggested we might counter these fears by making sure that potential patients are able to get a very clear understanding of the study and their expected involvement. Operationally, this led us to simplify the language used in describing the study: for example, the randomization process (including being clear about the possibility of being placed in the usual care group) and the intent of the interventions. We also built more time into the recruitment calls and in-person consenting process so that the patients are free to ask all their questions. In addition, field staff were trained in the ‘teach back’ method - asking patients to describe in their own words what the initiative was about and what their commitment was in order to determine areas that needed further clarification. Lastly, we incorporated the suggestion of focus group members that we stress the useful knowledge gained through research and how patients are the ones with the important information.

The intervention approach already stressed patient-centeredness in goal setting, but focus group findings led us to enhance staff training to further address the cultural issues that came up at our meetings. For example, to address the issue of trust or rather distrust in the medical care systems, we instituted staff discussions and practice sessions on how to maintain trust (for example, reflective listening, following up with community referrals when requested, addressing other clinical conditions if priority to the patient). Some of our focus group participants also expressed a sense of inevitability to their stroke experience and the possibility of having another stroke. These feelings seem to have been influenced partly by the view that chronic disease, hypertension, and diabetes are ‘passed down’ in the family. During our staff training, we discussed the implications of these beliefs (for example, how they may impact a person’s self-management behavior), how to identify these beliefs, and how to address them (for example, additional education). We also were guided by our participants to engage family and others who provided the patient’s primary social support if the patient indicated that this was desirable, to take the time to address medication administration and side effect questions and to explore patients’ attitudes and beliefs about medications - elements all integrated into the intervention approach. Additional direction for intervention staff on how to recognize depression and isolation risk was provided in the training as these were areas that our focus group informants felt could hinder adherence to recommended management guidelines and their recovery.

Two significant issues related to BP criteria for patient eligibility arose shortly before and after enrollment began, and both had potentially significant operational implications. A few weeks before launch the Program Advisory Committee for the Center for Stroke Disparities Solutions (CSDS) [43], parent of our study, advised the team to modify the BP eligibility screening process. Initially, the protocol was designed to determine patient eligibility based on a single face-to-face encounter in which the average of 3 SBP readings needed to be ≥ 140 mmHg after the patient passed other eligibility screening questions described above. The revised protocol calls for 2 face-to-face interviews in which 3 BP readings are taken and need to result in an average SBP of ≥ 140 mmHg in both interviews. Currently, there is no widely accepted scientific standard for enrolling patients in BP studies; some use historical data in medical records to identify potential study subjects [44,45], some enroll patients if they simply have a HTN diagnosis [46], some allow enrollment of those in pre-HTN stage [47], others adopt a higher SBP threshold of ≥ 150 mmHg at the time of enrollment [48] and some include patients with single BP measurements in the uncontrolled HTN range [49,50]. The intent of the more stringent screen employed by our trial is to increase the likelihood of enrollment of the intended target population - those with uncontrolled HTN. Adjustments to our workflow and budget were implemented, although it was unclear how many people would drop out after the second BP screening visit. Data from this revised screening process will help to inform future studies. As of 5 September 2014, of the 97 patients who passed the first BP screen, 65 (67%) met the SBP threshold at the second BP screen.

The second BP threshold issue that came up shortly after enrollment began derived from publication of the 2014 Evidence-Based Guideline for the Management of High Blood Pressure in Adults [51]. One of the 9 recommendations made is to treat to a goal of SBP < 150 mmHg for the general population aged 60 years and over, a departure from the prior guideline [52], which recommended the SBP goal of < 140 mmHg for all patients. None of the new recommendations addressed the goal SBP for post-stroke patients. We expect that around 60% of the patients enrolled in our study will be aged 60 years or over. Since there is a body of evidence showing the benefit of lower SBP for stroke risk reduction, and after a discussion with HTN specialists at the Center for Stroke Disparities Solutions, we did not alter our intervention approach to increase our SBP target to < 150 mmHg. However, there is some concern that primary care providers overseeing the long-term care of these patients may be influenced by these published guidelines and decline to more aggressively treat patients with SBP levels between 140 and 149 mmHg.

Results of this trial will provide important information on the design of interventions to address treatment gaps and disparities in recurrent stroke prevention for vulnerable black and Hispanic populations. The trial focuses on care transitions, a juncture when patients are particularly susceptible to adverse events. The interventions are innovative in adapting for stroke patients an established transitional care model shown to be effective for HF patients, pairing the professional NP with a HC, tailoring the interventions for black and Hispanic patients respectively, and placing primary emphasis on longer-term risk factor reduction and community reintegration rather than shorter-term transitional care outcomes.

## Trial status

Patient enrollment began in January 2014. Enrollment period is expected to be 30 months.

## Abbreviations

ADL: activities of daily living
AHA: American Heart Association
ASA: American Stroke Association
BECCI: Behavior Change Counseling Index
BP: blood pressure
BMQ: beliefs about medications
CSDS: Center for Stroke Disparities Solutions
CTI: Care Transitions Intervention®
CTIs: Community Transitions Interventions
EHR: electronic health record
EuroQol: health-related quality of life
FAB: Frontal Assessment Battery
FIML: full information maximum likelihood
GEE: generalized estimating equation
HC: health coach
HF: heart failure
HTN: hypertension
ICD-9: International Classification of Diseases, version 9
IPAQ: International Physical Activity Questionnaire
ITT: intent-to-treat
MINT: Motivational Interviewing Network of Trainers
NP: nurse practitioner
PCP: primary care provider
OASIS: Outcomes Assessment and Information Set
PACIC: Patient Assessment of Chronic Illness Care
RCT: randomized controlled trial
SBP: systolic blood pressure
TCM: Transitional Care Model
TIA: transient ischemic attack
UHC: usual home care
UHC + NP: usual home care complemented by a nurse practitioner
UHC + NP + HC: usual home care complemented by a nurse practitioner plus health coach
VNSNY: Visiting Nurse Service of New York
